# Telomere DNA damage signaling regulates cancer stem cell evolution, epithelial mesenchymal transition, and metastasis

**DOI:** 10.18632/oncotarget.20960

**Published:** 2017-09-16

**Authors:** Angelica M. Lagunas, Jianchun Wu, David L. Crowe

**Affiliations:** ^1^ University of Illinois Cancer Center, Chicago, IL, USA

**Keywords:** genomic instability, telomerase, kinase signaling, differentiation

## Abstract

Chromosome ends are protected by telomeres that prevent DNA damage response and degradation. When telomeres become critically short, the DNA damage response is activated at chromosome ends which induces cellular senescence or apoptosis. Telomeres are protected by the double stranded DNA binding protein TRF2 and maintained by telomerase or a recombination based mechanism known as alternative lengthening of telomeres (ALT). Telomerase is expressed in the basal layer of the epidermis, and stem cells in epidermis have longer telomeres than proliferating populations. Stem cell expansion has been associated with epithelial-mesenchymal transition (EMT) in cancer. EMT is a critical process in cancer progression in which cells acquire spindle morphology, migrate from the primary tumor, and spread to distant anatomic sites. Our previous study demonstrated that loss of TRF2 expression observed in human squamous cell carcinomas expanded metastatic cancer stem cells during mouse skin carcinogenesis. To determine if telomerase inhibition could block the TRF2-null mediated expansion of metastatic clones, we characterized skin carcinogenesis in a conditional TRF2/Terc double null mutant mouse. Loss of TRF2 and Terc expression resulted in telomere DNA damage, severely depleted CD34 + and Lgr6+ cancer stem cells, and induced terminal differentiation of metastatic cancer cells. However a novel cancer stem cell population evolved in primary tumors exhibiting genomic instability, ALT, and EMT. Surprisingly we discovered that metastatic clones evolved prior to histopathologic onset of primary tumors. These results have important implications for understanding the evolution and treatment of metastatic cancer.

## INTRODUCTION

Chromosome ends are protected by telomeres that prevent DNA damage response and degradation [1]. Telomeres form a large duplex loop mediated by single-strand invasion of a G-rich overhang [2, 3]. When telomeres become critically short the DNA damage response is activated at chromosome ends [4], which induces cellular senescence or apoptosis.

The telomeric shelterin complex contains the double stranded DNA binding protein TRF2 [1]. Dominant negative TRF2 induced end to end chromosomal fusions of telomeric DNA [5]. Cells expressing the dominant negative TRF2 protein undergo senescence or apoptosis. Programmed cell death was mediated by ATM kinase and p53 consistent with DNA damage checkpoint activation [6–8]. Nonhomologous end joining of telomeres was dependent on DNA ligase IV [9]. These studies demonstrate the importance of TRF2 in regulating DNA damage response at telomeres.

Cellular subpopulations can stabilize their telomeres and continue proliferation by upregulation of telomerase [10–12]. Telomerase extends telomeres using its Terc RNA template [13, 14]. Telomerase overexpression can inhibit telomeric DNA damage response and immortalize cultured cells. Given the positive effects of telomerase on telomere length and cellular proliferation, telomerase activity is commonly upregulated in cancer cell lines and primary tumors [15]. In Terc-null mutant mice, cells with short telomeres exhibit DNA damage response followed by chromosomal fusions, aneuploidy, and apoptosis [11, 12]. Telomerase-negative immortal cells exhibited significant heterogeneity of telomere length, suggesting an alternative mechanism of telomere lengthening [16]. This alternative lengthening of telomeres (ALT) is a recombination-based mechanism associated with the formation of ALT-associated promyelocytic leukemia bodies (APB; 17). Replication products of this pathway such as circular C-rich strands are present in ALT cells [18].

Alterations in telomere length regulation have profound effects on stem cells in the epidermis [19, 20]. Stem cells have longer telomeres than proliferating populations found in the epidermis [21]. In mammalian epidermis, important stem cell populations reside in the adult hair follicle [22–24]. These slowly cycling CD34+ or Lgr6+ cells respond to external stimuli by increased cell division and migration, and they are capable of regenerating components of the epidermis [25–27]. Loss of telomerase activity inhibited proliferation and mobilization of stem cells and impaired hair growth [28], whereas telomerase overexpression caused transition to anagen with robust hair growth [29–30]. Telomerase is also expressed in the basal layer of the epidermis, and its overexpression in this tissue increases tumor formation [31, 32].

Cancer stem cell expansion has been associated with epithelial-mesenchymal transition (EMT; 33). EMT is a critical process in cancer progression in which cells acquire spindle morphology, migrate from the primary tumor, and spread to distant anatomic sites. Cancer cells undergoing EMT have characteristic changes in gene expression which regulate morphology and invasion. These changes include increased Snail, Twist, and vimentin levels and decreased expression of epithelial genes such as E-cadherin, p63, and keratins. EMT has been associated with poor clinical prognosis including metastasis and chemotherapy resistance.

TRF2 expression is reduced in many types of cancer [34–36]. We previously demonstrated that loss of TRF2 expression depletes CD34+ stem cells in epidermis [37]. However, these stem cell depleted mice exhibit significant cancer stem cell expansion and metastasis during carcinogenesis due to telomere DNA damage response, genomic instability, and aneuploidy. To determine if telomerase inhibition which is being tested in cancer clinical trials could block the TRF2-null mediated expansion of metastatic clones, we characterized skin carcinogenesis in a conditional TRF2/Terc double null mutant mouse. Our results have important implications for understanding the evolution and treatment of metastatic cancer.

## RESULTS

**Figure 1:**
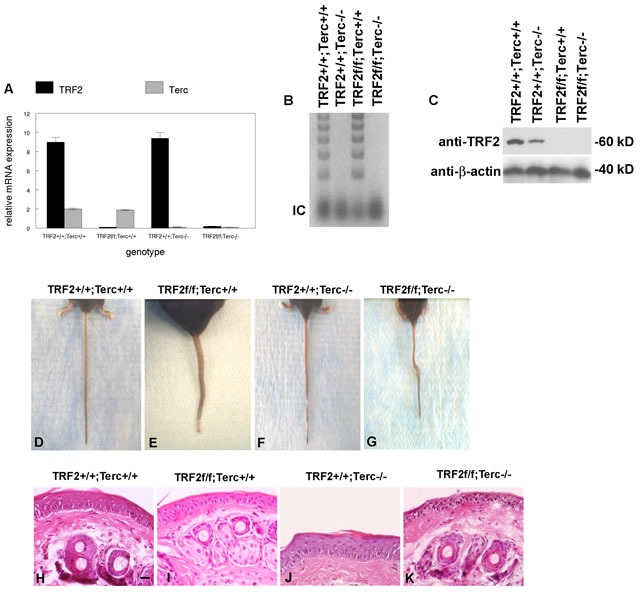
TRF2/Terc double null mutant mice exhibit features of stem cell depletion in epidermis **A.** TRF2 mRNA and Terc RNA expression in K14Cre;TRF2+/+;Terc+/+, K14Cre;TRF2f/f;Terc+/+, K14Cre;TRF2+/+;Terc-/-, and K14Cre;TRF2f/f;Terc-/- epidermis is shown by qRT-PCR. Error bars represent SEM. **B.** Telomerase activity in epidermis from the indicated genotypes is shown by TRAP assay. Internal amplification controls are shown. A representative gel is shown. **C.** TRF2 protein expression in epidermis from the indicated genotypes is shown by western blot. β-actin expression is shown as the gel loading control for each lane. Representative blots are shown. **D.**-**G.** TRF2/Terc double null mutant mice exhibit a phenotype similar to the *crinkled* mouse. Representative photographs of mouse tails from the indicated genoptypes are shown. **H.**-**K.** Skin histopathology of the indicated genotypes is shown by H&E staining. Scale bar = 10 μm. Representative photomicrographs are shown.

To determine if telomerase inhibition could block TRF2-null mediated cancer stem cell expansion in epidermis, we bred the K14Cre;TRF2f/f mouse to G1 Terc-/- animals. K14Cre;TRF2f/f;Terc-/- mice were born at the expected mendelian frequencies and were grossly normal with respect to appearance, weight, and behavior. Epidermis from K14Cre;TRF2f/f;Terc-/- mice lacked both TRF2 mRNA and protein expression (Figure 1A, 1C). Terc RNA expression and telomerase activity also was undetectable in K14Cre;TRF2f/f;Terc-/- epidermis (Figure 1A, 1B). By eight weeks of age, K14Cre;TRF2f/f;Terc-/- mice exhibited prominent tail bends or loops of up to 90^0^ (Figure 1G). This phenotype was not observed in K14Cre;TRF2+/+;Terc+/+ or K14Cre;TRF2+/+;Terc-/- but was observed to a lesser extent in K14Cre;TRF2f/f;Terc+/+ mice (Figure 1D-1F). Histopathologic examination of skin from K14Cre;TRF2f/f;Terc-/- mice revealed reduced epidermal thickness with absence of suprabasal layers compared to K14Cre;TRF2+/+;Terc+/+ and K14Cre;TRF2+/+;Terc-/- animals (Figure 1H, 1J, 1K). Intermediate reduction in epidermal thickness was observed in K14Cre;TRF2f/f;Terc+/+ mice (Figure 1I). No significant differences in the morphology of dermal appendages such as hair follicles or sebaceous glands were noted. These results indicated that the TRF2/Terc double null mutation produces phenotypes consistent with epidermal stem cell depletion.

**Figure 2:**
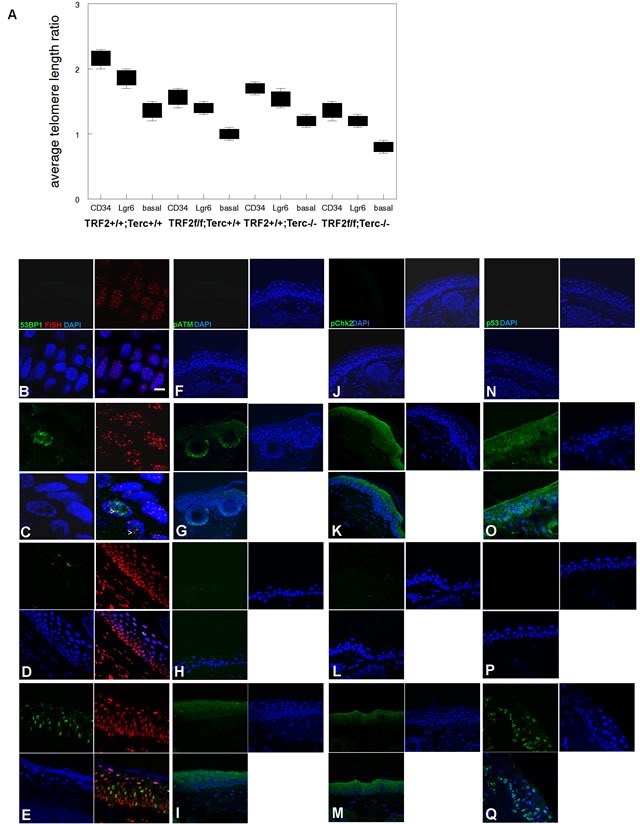
TRF2/Terc double null mutant mice exhibit DNA damage response at short telomeres in epidermis **A.** Average telomere length ratios in CD34+ stem, Lgr6+ stem, and basal cells from K14Cre;TRF2+/+;Terc+/+, K14Cre;TRF2f/f;Terc+/+, K14Cre;TRF2+/+;Terc-/-, and K14Cre;TRF2f/f;Terc-/- epidermis were determined by qPCR. Error bars represent SEM. Co-localization of 53BP1 (shown by immunofluorescence, AlexaFluor 488) at telomeres (shown by fluorescence in situ hybridization, Cy3) in histopathologic sections from K14Cre;TRF2+/+;Terc+/+ **B.**, K14Cre;TRF2f/f;Terc-/- **C.**, K14Cre;TRF2+/+;Terc-/- **D.**, and K14Cre;TRF2f/f;Terc+/+ **E.** epidermis is shown. Nuclei are counterstained with DAPI. Scale bar = 5 μm. Phospho-ATM expression in histopathologic sections from K14Cre;TRF2+/+;Terc+/+ **F.**, K14Cre;TRF2f/f;Terc-/- **G.**, K14Cre;TRF2+/+;Terc-/- **H.**, and K14Cre;TRF2f/f;Terc+/+ **I.** epidermis. Phospho-Chk2 expression in histopathologic sections from K14Cre;TRF2+/+;Terc+/+ **J.**, K14Cre;TRF2f/f;Terc-/- **K.**, K14Cre;TRF2+/+;Terc-/- **L.**, and K14Cre;TRF2f/f;Terc+/+ **M.** epidermis. p53 protein expression in histopathologic sections from K14Cre;TRF2+/+;Terc+/+ **N.**, K14Cre;TRF2f/f;Terc-/- **O.**, K14Cre;TRF2+/+;Terc-/- **P.**, and K14Cre;TRF2f/f;Terc+/+ **Q.** epidermis. Representative sections are shown.

K14Cre;TRF2f/f;Terc-/- epidermis exhibited dramatic telomere shortening in both stem and basal cells indicative of telomere DNA damage response (ATLR 1.4 vs. 2.2 for CD34+ stem cells, 1.3 vs. 1.8 for Lgr6+ stem cells, 0.8 vs. 1.4 for basal cells; Figure 2A). K14Cre;TRF2f/f;Terc-/- and K14Cre;TRF2+/+;Terc-/- epidermis exhibited intermediate telomere shortening. We characterized telomere DNA damage response in the epidermis of K14Cre;TRF2f/f;Terc-/- and K14Cre;TRF2+/+;Terc+/+ mice. Cells with greater than 4 telomere DNA damage foci were considered positive in this analysis. K14Cre;TRF2f/f;Terc-/- epidermis exhibited increased 53BP1 DNA damage foci at telomeres compared to K14Cre;TRF2+/+;Terc+/+ epidermis (31% vs. 0.1%; *P* < 10^−5^; Figure 2B, 2C). Colocalization of 53BP1 foci at telomeres was observed to lesser extents in K14Cre;TRF2+/+;Terc-/- (9%; *P* < 0.001; Figure 2D) and K14Cre;TRF2f/f;Terc+/+ (19%; *P* < 0.005; Figure 2E) epidermis. Phospho-ATM expression was strongly induced in both basal and suprabasal cells, and in hair follicles of K14Cre;TRF2f/f;Terc-/- skin compared to the K14Cre;TRF2+/+;Terc+/+ genotype (79% vs. 0.1%; *P* < 10^−6^; Figure 2F, 2G). Lesser pATM induction was observed in K14Cre;TRF2f/f;Terc+/+ epidermis (54%; Figure 2I), and background expression of phospho-ATM was observed in K14Cre;TRF2+/+;Terc-/- epidermis (Figure 2H). Phospho-Chk2 expression was strongly induced in both basal and suprabasal cells of K14Cre;TRF2f/f;Terc-/- compared to K14Cre;TRF2+/+;Terc+/+ epidermis (86% vs. 0.1%; *P* < 10^−6^; Figure 2J, 2K). Lesser pChk2 induction was observed in K14Cre;TRF2f/f;Terc+/+ epidermis (62%; Figure 2M), and background pChk2 expression was observed in K14Cre;TRF2+/+;Terc+/+ epidermis (Figure 2L). p53 expression was induced in K14Cre;TRF2f/f;Terc-/- compared to K14Cre;TRF2+/+;Terc+/+ epidermis (89% vs. 0.2%; *P* < 10^−7^; Figure 2N, 2O). Lesser p53 induction was observed in K14Cre;TRF2f/f;Terc+/+ epidermis (26%; Figure 2Q), and background p53 expression was observed in K14Cre;TRF2+/+;Terc-/- epidermis (Figure 2P). We noted both cytoplasmic and nuclear p53 expression in K14Cre;TRF2f/f;Terc-/- but not K14Cre;TRF2f/f;Terc+/+ epidermis, which may be due to higher p53 expression induced by the telomere DNA damage response in the double null mutant mouse. These results indicate that loss of both TRF2 expression and telomerase activity induces telomeric DNA damage signaling and telomere shortening in mouse epidermis.

**Figure 3:**
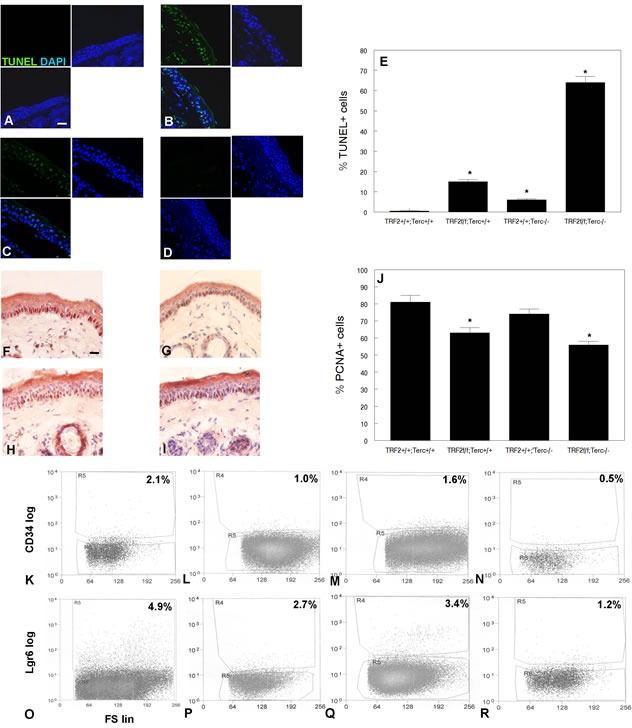
TRF2/Terc double null mutant mice exhibit increased apoptosis, decreased proliferation, and reduction in the CD34+ and Lgr6+ stem cell populations in the epidermis Apoptotic cells were detected in K14Cre;TRF2+/+;Terc+/+ **A.**, K14Cre;TRF2f/f;Terc-/- **B.**, K14Cre;TRF2f/f;Terc+/+ **C.**, and K14Cre;TRF2+/+;Terc-/- **D.** epidermis by TUNEL assay. Scale bar = 5 μm. **E.** Quantitation of TUNEL+ cells in epidermis of indicated genotypes. Error bars represent SEM. Asterisks indicate statistically significant differences. Proliferating cells were detected in K14Cre;TRF2+/+;Terc+/+ **F.**, K14Cre;TRF2f/f;Terc-/- **G.**, K14Cre;TRF2f/f;Terc+/+ **H.**, and K14Cre;TRF2+/+;Terc-/- **I.** epidermis by PCNA immunohistochemistry. **J.** Quantitation of PCNA+ cells. Error bars indicate SEM. Asterisks indicate statistically significant differences. FACS analysis of CD34+ stem cells from K14Cre;TRF2+/+;Terc+/+ **K.**, K14Cre;TRF2f/f;Terc+/+ **L.**, K14Cre;TRF2+/+;Terc-/- **M.**, and K14Cre;TRF2f/f;Terc-/- **N.** epidermis. FACS analysis of Lgr6+ stem cells from K14Cre;TRF2+/+;Terc+/+ **O.**, K14Cre;TRF2f/f;Terc+/+ **P.**, K14Cre;TRF2+/+;Terc-/- **Q.**, and K14Cre;TRF2f/f;Terc-/- **R.** epidermis. CD34 and Lgr6 fluorescence is shown by log scale (y axis) and forward scatter is shown by linear scale (x axis). The percentage of positive cells in each group is shown.

To determine the effect of this telomeric DNA damage signaling at the cellular level, we first examined programmed cell death in K14Cre;TRF2f/f;Terc-/- and control epidermis. K14Cre;TRF2f/f;Terc-/- epidermis exhibited significantly increased numbers of TUNEL+ cells compared to control skin (64% vs. 1.1%; P < 0.00001; Figure 3A, 3B, 3E). Intermediate and low apoptotic cell fractions were observed in K14Cre;TRF2f/f;Terc+/+ (15%) and K14Cre;TRF2+/+;Terc-/- (6%) epidermis (Figure 3C, 3D, 3E). K14Cre;TRF2f/f;Terc-/- basal cells exhibited significantly decreased proliferation index as shown by PCNA immunohistochemistry compared to K14Cre;TRF2+/+;Terc+/+ epidermis (54% vs. 81%; *P* < 0.03; Figure 3F, 3G, 3J). K14Cre;TRF2f/f;Terc+/+ (61%) and K14Cre;TRF2+/+;Terc-/- (74%) basal cells exhibited intermediate reductions of proliferating cells (Figure 3H-3J). We sorted CD34+ and Lgr6+ epidermal stem cell populations from K14Cre;TRF2f/f;Terc-/- and K14Cre;TRF2+/+;Terc+/+ epidermis by flow cytometry. FACS analysis of CD34+ stem cells from K14Cre;TRF2f/f;Terc-/- epidermis showed significant depletion of this population compared to K14Cre;TRF2+/+;Terc+/+ skin (0.5% vs. 2.1%; *P* < 0.002; Figure 3K, 3N). Intermediate depletion of CD34+ stem cells was observed in K14Cre;TRF2f/f;Terc+/+ (1.0%) and K14Cre;TRF2+/+;Terc-/- (1.6%) epidermis (Figure 3L, 3M). FACS analysis of Lgr6+ stem cells from K14Cre;TRF2f/f;Terc-/- epidermis also showed significant depletion of this population compared to K14Cre;TRF2+/+;Terc+/+ skin (1.2% vs. 4.9%; *P* < 0.0004; Figure 3O, 3R). Intermediate depletion of Lgr6+ stem cells was observed in K14Cre;TRF2f/f;Terc+/+ (2.7%) and K14Cre;TRF2+/+;Terc-/- (3.4%) epidermis (Figure 3P, 3Q). These results indicate that loss of both TRF2 expression and telomerase activity increases apoptosis, decreases proliferation, and depletes distinct stem cell populations in mouse epidermis.

**Figure 4:**
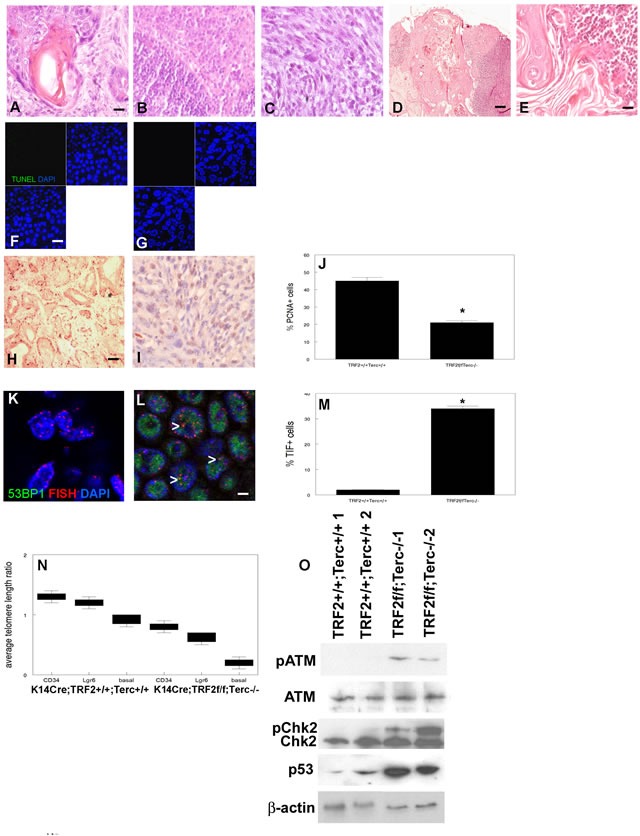
TRF2/Terc double null mutation induces DNA damage response resulting in terminal differentiation of metastatic tumors Histopathology of primary SCC from K14Cre;TRF2+/+;Terc+/+ **A.** and K14Cre;TRF2f/f;Terc-/- **C.** mice is shown by H&E staining. Scale bar = 10 μm. Histopathology of metastatic SCC from K14Cre;TRF2+/+;Terc+/+ **B.** and K14Cre;TRF2f/f;Terc-/- **D.**, **E.** mice is shown by H&E staining. Scale bar in (D) = 50 μm. Apoptotic cells in primary SCC from K14Cre;TRF2+/+;Terc+/+ **F.** and K14Cre;TRF2f/f;Terc-/- **G.** mice are shown by TUNEL assay. Scale bar = 10 μm. Proliferating cells were detected in K14Cre;TRF2+/+;Terc+/+ **H.** and K14Cre;TRF2f/f;Terc-/- **I.** primary SCC by PCNA immunohistochemistry and quantitated in **J.**. Error bars indicate SEM. Asterisk indicates statistically significant difference. Co-localization of 53BP1 (shown by immunofluorescence, AlexaFluor 488) at telomeres (shown by fluorescence in situ hybridization, Cy3) in histopathologic sections from K14Cre;TRF2+/+;Terc+/+ **K.** and K14Cre;TRF2f/f;Terc-/- **L.** primary SCC and quantitated in **M.** Error bars indicate SEM. Nuclei were counterstained with DAPI. Scale bar = 5 μm. **N.** Average telomere length ratios in CD34+ stem, Lgr6+ stem, and basal cells from K14Cre;TRF2+/+;Terc+/+ and K14Cre;TRF2f/f;Terc-/- primary SCC were determined by qPCR. Error bars represent SEM. **O.** Phospho-ATM, phospho-Chk2, and p53 expression in K14Cre;TRF2+/+;Terc+/+ and K14Cre;TRF2f/f;Terc-/- primary SCC is shown by western blot. β-actin expression was used to control for equal loading of each lane. Representative blots are shown.

We next examined the effects of the TRF2/Terc double null mutation on epidermal carcinogenesis. We previously characterized epidermal carcinogenesis in Terc null and TRF2 null mice (37, 38; Supplementary Figure 1). Our DMBA protocol resulted in SCC in all treated mice; there were no significant differences in the number of tumors between experimental and control groups. K14Cre;TRF2f/f;Terc+/+ SCC exhibited increased latency (22 vs. 18 weeks in K14Cre;TRF2+/+;Terc+/+; *P* < 0.03; Supplementary Figure 1A). Tumor latency of K14Cre;TRF2f/f;Terc-/- SCC was decreased compared to TRF2 null mutant cancers (17 weeks; *P* < 0.05). Control, Terc null, and TRF2 null mice developed primarily well differentiated primary SCC as shown in Figure 4A and Supplementary Figure 1B, C. Lymph node metastases were moderately differentiated in K14Cre;TRF2+/+;Terc+/+, K14Cre;TRF2+/+;Terc-/-, and K14Cre;TRF2f/f;Terc+/+ genotypes as shown in Figure 4B and Supplementary Figure 1D, E. Surprisingly K14Cre;TRF2f/f;Terc-/- primary tumors were poorly differentiated SCC (Figure 4C). In striking contrast to the primary tumors, all K14Cre;TRF2f/f;Terc-/- metastatic SCC were terminally differentiated with no evidence of proliferating cancer cells (Figure 4D, 4E). No terminally differentiated metastatic lesions were observed in the control group. We did not observe significant differences in apoptotic cell fraction between K14Cre;TRF2+/+;Terc+/+ and K14Cre;TRF2f/f;Terc-/- primary SCC (0.1%; Figure 4F, 4G). Apoptotic cell fraction was significantly increased in Terc null (5%) and TRF2 null (15%) SCC (Supplementary Figure 1F-H; *P* < 0.001). Cell proliferation was significantly decreased in Terc null, TRF2 null, and K14Cre;TRF2f/f;Terc-/- cancers compared to control SCC as determined by PCNA immunohistochemistry (9%, 5%, and 20% vs. 45%; P < 0.001; Figure 4H-4J; Supplementary Figure 1I-K). Telomere DNA damage foci were significantly increased in Terc null, TRF2 null, and K14Cre;TRF2f/f;Terc-/- compared to control SCC (10%, 26%, and 34% vs. 2%; *P* < 0.0002; Figure 4K-4M; Supplementary Figure 1L-N). Due to severe depletion of cancer stem cells in double null tumors we used a quantitative PCR method to measure relative telomere length. Relative telomere length was significantly reduced in stem and basal cells from Terc null (0.9-0.6), TRF2 null (0.7-0.4), and K14Cre;TRF2f/f;Terc-/- (0.7-0.2) compared to control SCC (1.3-0.8; *P* < 0.0001; Figure 4N; Supplementary Figure 2A). DNA damage signaling proteins were significantly activated in TRF2 null and K14Cre;TRF2f/f;Terc-/- compared to control SCC (P < 0.004; Figure 4O; Supplementary Figure 2B). These results indicate that poorly differentiated K14Cre;TRF2f/f;Terc-/- SCC exhibit highest levels of telomere DNA damage signaling, telomere shortening, and reduced proliferation. These highest levels of telomere DNA damage signaling correlated with terminal differentiation of metastatic K14Cre;TRF2f/f;Terc-/- SCC.

**Figure 5:**
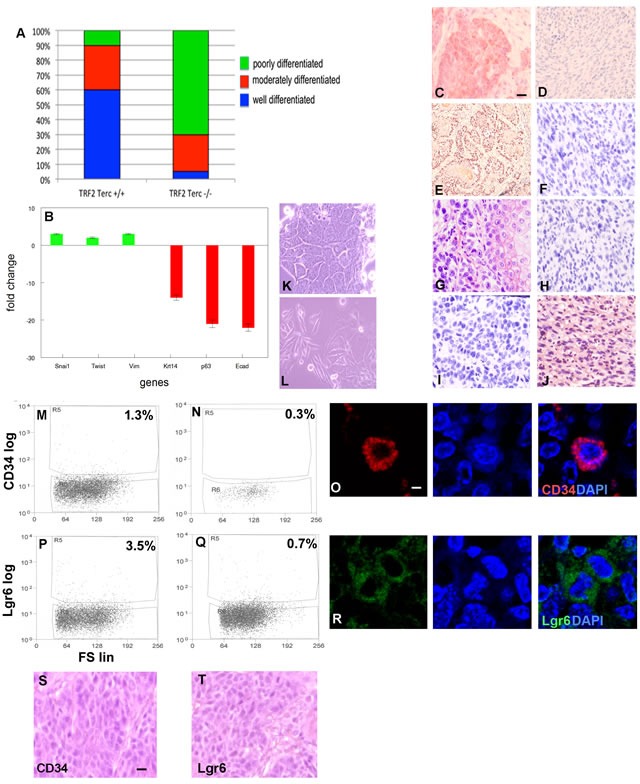
TRF2/Terc double null mutation induces EMT despite pronounced depletion of tumorigenic CD34+ and Lgr6+ cancer stem cells **A.** Percentage of histopathologically confirmed well, moderate, and poorly differentiated primary SCC in K14Cre;TRF2+/+;Terc+/+ and K14Cre;TRF2f/f;Terc-/- mice. **B.** Fold change in expression of EMT (Snai1, Twist, Vim) and epithelial differentiation (Krt14, p63, Ecad) mRNAs in K14Cre;TRF2f/f;Terc-/- compared to K14Cre;TRF2+/+;Terc+/+ SCC. Keratin 14 **C.**, **D.** p63 **E.**, **F.** E-cadherin **G.**, **H.**, and vimentin **I.**, **J.** protein expression in K14Cre;TRF2+/+;Terc+/+ (left) and K14Cre;TRF2f/f;Terc-/- (right) SCC by immunohistochemistry. Scale bar = 10 μm. Cellular morphology of K14Cre;TRF2+/+;Terc+/+ **K.** and K14Cre;TRF2f/f;Terc-/- **L.** SCC in monolayer cultures is shown by phase contrast microscopy. Representative photomicrographs are shown. FACS analysis of CD34+ cancer stem cells from K14Cre;TRF2+/+;Terc+/+ **M.** and K14Cre;TRF2f/f;Terc-/- **N.** SCC. Fluorescence is shown by log scale (y axis) and forward scatter is shown by linear scale (x axis). The percent positive cells in each group are shown. **O.** Immunofluorescent localization of CD34+ cancer stem cells in primary SCC. Nuclei were counterstained with DAPI. Scale bar = 2 μm. FACS analysis of Lgr6+ cancer stem cells from K14Cre;TRF2+/+;Terc+/+ **P.** and K14Cre;TRF2f/f;Terc-/- **Q.** SCC. **R.** Immunofluorescent localization of Lgr6+ cancer stem cells in primary SCC. Reconstituted SCC from subcutaneous transplantation of sorted CD34+ **S.** and Lgr6+ **T.** cancer stem cells in immunodeficient mice is shown by H&E staining. Scale bar = 10 μm. Representative photomicrographs are shown.

Seventy percent of K14Cre;TRF2f/f;Terc-/- primary SCC exhibited poorly differentiated histopathology with spindle cell morphology compared to only 10% of control cancers (Figure 5A), suggesting that double null tumors undergo EMT. Gene expression analysis demonstrated 2-3 fold induction of EMT markers Snail, Twist, and vimentin in K14Cre;TRF2f/f;Terc-/- SCC (*P* < 0.04; Figure 5B). Dramatic reductions in expression of stratified epithelial markers keratin 14, p63, and E-cadherin were also observed in K14Cre;TRF2f/f;Terc-/- SCC (9-23 fold; *P* < 0.003). EMT was not observed in Terc null and TRF2 null SCC (Supplementary Figure 3A). We confirmed keratin 14 (Figure 5C, 5D), p63 (Figure 5E, 5F), E-cadherin (Figure 5G, 5H), and vimentin (Figure 5I, 5J) protein expression by immunohistochemistry in K14Cre;TRF2f/f;Terc-/- and control SCC. We grew K14Cre;TRF2f/f;Terc-/- and control SCC cells in monolayer culture. Control SCC exhibited characteristic cobblestone colonies in culture, while double null cancer cells retained spindle cell morphology (Figure 5K, 5L). K14Cre;TRF2f/f;Terc-/- cancer cells were more invasive that those of the other three genotypes using in vitro invasion analyses (*P* < 0.003; Supplementary Figure 3B-E). We sorted CD34+ cancer stem cells from K14Cre;TRF2f/f;Terc-/- and control SCC by flow cytometry. Double null SCC exhibited severe depletion of this cancer stem cell population (0.3% vs. 1.3%; *P* < 0.04; Figure 5M, 5N) which was significantly greater than depletion observed in Terc null tumors (1.0%; *P* < 0.03; Supplementary Figure 3F). TRF2 null SCC exhibited marked expansion of CD34+ cancer stem cells as previously reported (37; 8.0%; *P* < 0.003; Supplementary Figure 3G). We demonstrated CD34+ cancer stem cells in these tumors by immunofluorescence microscopy (Figure 5O). We also sorted Lgr6+ cancer stem cells from K14Cre;TRF2f/f;Terc-/- and control SCC by flow cytometry. Double null SCC exhibited severe depletion of this cancer stem cell population (3.5% vs. 0.7%; *P* < 0.009; Figure 5P, 5Q) which was significantly greater than that observed in Terc null (3.3%) and TRF2 null (0.9%) tumors (Supplementary Figure 3H, I). We demonstrated Lgr6+ cancer stem cells in these tumors by immunofluorescence microscopy (Figure 5R). Subcutaneous transplantation of 10^3^ CD34+ or Lgr6+ cells to immunocompromised mice resulted in poorly differentiated SCC (Figure 5S, 5T). These results indicate that K14Cre;TRF2f/f;Terc-/- SCC undergo EMT with severe cancer stem cell depletion.

**Figure 6:**
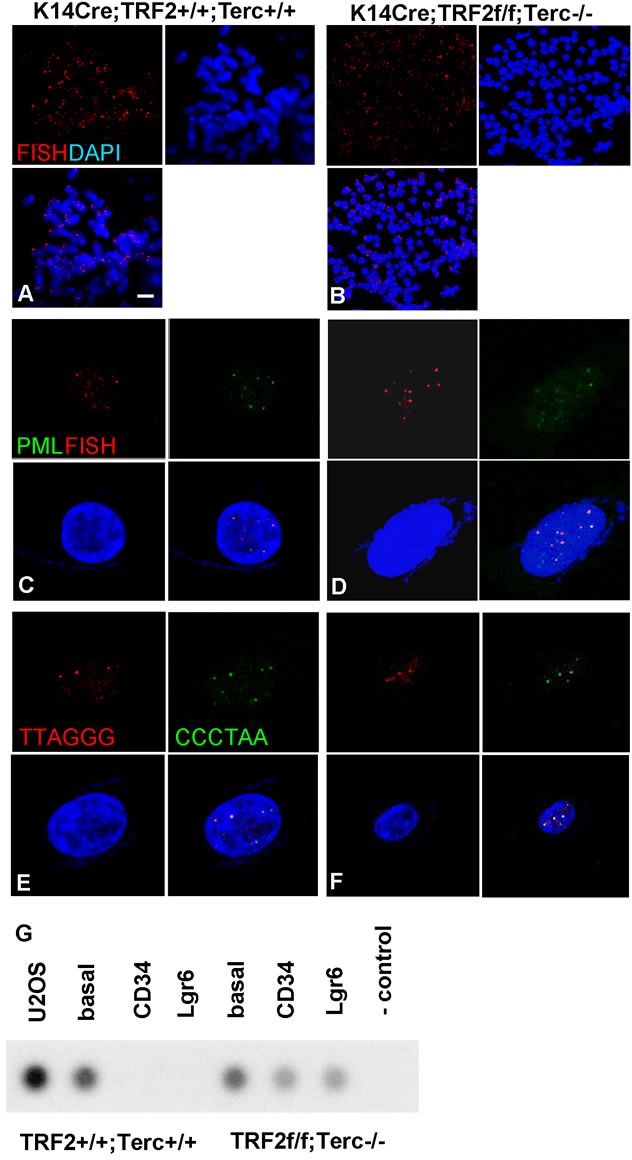
TRF2/Terc double null mutation induces chromosomal instability and ALT in primary SCC **A.** Telomeres are shown by FISH (Cy3) on metaphase chromosomal spreads of SCC cells from K14Cre;TRF2+/+;Terc+/+ (A) and K14Cre;TRF2f/f;Terc-/- **B.** mice. Chromosomes were counterstained with DAPI. Scale bar = 2 μm. APBs (PML protein shown by immunofluorescence, AlexaFluor 488) at telomeres (shown by fluorescence in situ hybridization, Cy3) in K14Cre;TRF2+/+;Terc+/+ **C.** and K14Cre;TRF2f/f;Terc-/- **D.** primary tumors are shown. Nuclei were counterstained with DAPI. ALT-associated sister chromatid exchange in K14Cre;TRF2+/+;Terc+/+ **E.** and K14Cre;TRF2f/f;Terc-/- **F.** SCC cells is shown by CO-FISH. Co-localization of DNA strand specific signals (Cy3, FITC) indicate sister chromatid exchange. Representative photomicrographs are shown. **G.** Telomeric circular DNA in sorted CD34+, Lgr6+, and CD34-Lgr6- basal cells from K14Cre;TRF2+/+;Terc+/+ and K14Cre;TRF2f/f;Terc-/- SCC. U2OS cells were used as the positive control, and reactions without genomic DNA or polymerase were used as the negative control.

To determine if combined TRF2 and Terc deficiency resulted in genomic instability and telomere DNA damage response leading to EMT, we performed telomere FISH on metaphase chromosomal spreads from K14Cre;TRF2+/+;Terc+/+ and K14Cre;TRF2f/f;Terc-/- SCC cells. Double null tumors exhibited extreme aneuploidy (modal chromosome number = 151 vs. 49 for K14Cre;TRF2+/+;Terc+/+ SCC; *P* < 0.00003) and dramatic shortening of most chromosomes (Figure 6A, 6B) which was significantly greater than that observed in Terc null and TRF2 null cancers (52 and 77; *P* < 0.004; Supplementary Figure 4A, 4B). In K14Cre;TRF2f/f;Terc-/- SCC, most chromosomal ends failed to exhibit telomere signal as detected by FISH (69% signal free ends vs. 11% for K14Cre;TRF2+/+;Terc+/+ SCC; *P* < 0.004). Given that some chromosomal ends displayed telomere signal by FISH, we wanted to determine if telomeres in K14Cre;TRF2f/f;Terc-/- SCC were maintained by the ALT pathway. We examined an established marker of ALT, namely ALT associated PML bodies (APB). Surprisingly PML protein colocalized at telomeres in both K14Cre;TRF2f/f;Terc-/- (51%) and K14Cre;TRF2+/+;Terc+/+ (45%) SCC cells (Figure 6C, 6D) which was also observed in Terc null (43%) and TRF2 null (49%) cancers (Supplementary Figure 4C, 4D). These differences were not statistically significant (*P* < 0.2). To more rigorously test for ALT pathway activation in K14Cre;TRF2f/f;Terc-/- and K14Cre;TRF2+/+;Terc+/+ SCC cells, we examined telomere sister chromatid exchange (TSCE) using chromosome orientation-fluorescence in situ hybridization. TSCE was detected in both K14Cre;TRF2f/f;Terc-/- (54%) and K14Cre;TRF2+/+;Terc+/+ (44%) SCC cells (Figure 6E, 6F) which was also observed in Terc null (41%) and TRF2 null (48%) cancers (Supplementary Figure 4E, 4F). As a third test of ALT pathway activation, we sorted CD34+, Lgr6+, and CD34-Lgr6- cells from K14Cre;TRF2f/f;Terc-/- and K14Cre;TRF2+/+;Terc+/+ SCC. Genomic DNA from these populations was subjected to analysis of telomeric circular DNA, a recognized marker of the ALT pathway. Telomeric circular DNA was detected in all three sorted cell populations in K14Cre;TRF2f/f;Terc-/- SCC cells, and in CD34-Lgr6- basal cells from K14Cre;TRF2+/+;Terc+/+ cancers (Figure 6G). Telomeric circular DNA also was detected in Terc null and TRF2 null sorted SCC cells (Supplementary Figure 4G). These results indicate that cancer stem cells with telomere DNA damage exhibit ALT activity, which was common to basal cells of cancers from all genotypes.

**Figure 7:**
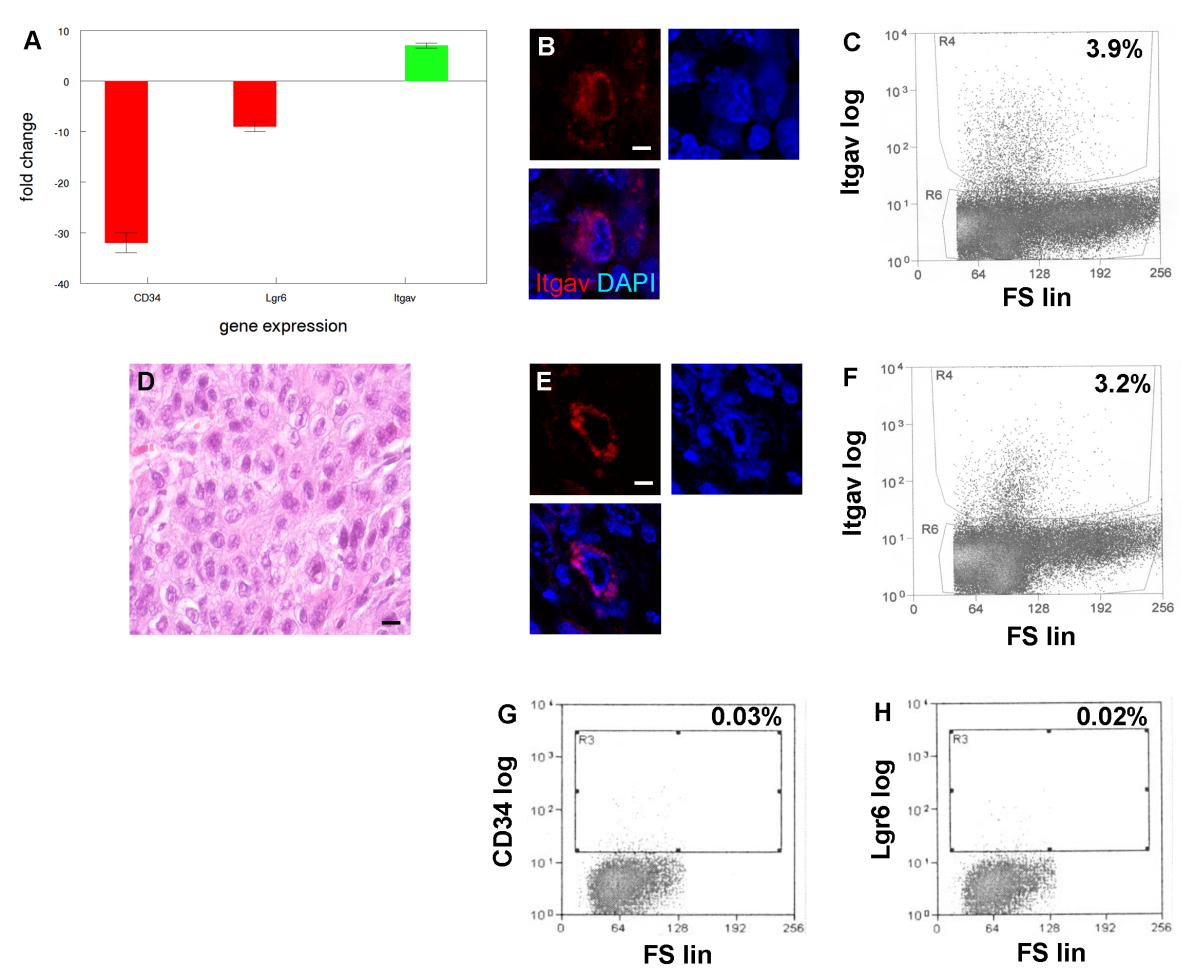
A new tumor initiating cell population with limited differentiation capacity from SCCs with EMT phenotype **A.** Increased expression of integrin α_V_ (Itgav) in cells from SCC with EMT phenotype. qRT-PCR shows decreased expression of known cancer stem cell markers CD34 and Lgr6 in Itgav+ cancer cells. Experiments were performed three times with similar results. Error bars indicate SEM. **B.** Itgav+ cancer cell (red) in SCC with EMT phenotype. Nuclei were counterstained with DAPI. Scale bar = 5 μm. A representative section is shown. **C.** Flow cytometric sorting of Itgav+ cancer cells from SCC with EMT phenotype. Itgav log and forward scatter linear (FS lin) scales are shown. A representative sort is shown. **D.** H&E stained section of poorly differentiated SCC arising from transplanted Itgav+ cancer cells. **E.** Itgav+ cancer cell (red) in transplanted SCC. Scale bar = 5 μm. **F.** Flow cytometric sorting of Itgav+ cancer cells from transplanted SCC. Itgav+ cancer cells fail to regenerate CD34+ or Lgr6+ cancer stem cells. CD34+ **G.** and Lgr6+ **H.** cancer stem cells were sorted from transplanted SCC by flow cytometry.

Both CD34+ and Lgr6+ cancer stem cell populations from K14Cre;TRF2f/f;Terc-/- SCCs were tumorigenic in our transplantation experiments. However these cells comprised a small fraction of total cells in the tumors, and as few as 10^3^ CD34-Lgr6- basal cells formed cancers when serially transplanted suggesting that additional tumorigenic populations were present in these cancers. Gene expression analysis of the CD34-Lgr6- cancer cell population revealed 7 fold increased expression of integrin α_V_ (Itgav; Figure 7A; *P* < 0.02). These cells exhibited dramatically reduced expression of CD34 (32 fold reduction; P < 0.0001) and Lgr6 (9 fold reduction; *P* < 0.003; Figure 7A). Itgav+ cells were identified in K14Cre;TRF2f/f;Terc-/- but not K14Cre;TRF2+/+;Terc+/+, K14Cre;TRF2f/f;Terc+/+, nor K14Cre;TRF2+/+;Terc-/- primary tumors by immunofluorescence microscopy (Figure 7B) and comprised 3.9% of cells in double null cancers as shown by flow cytometry (Figure 7C). When we subcutaneously transplanted 10^3^ Itgav+ cancer cells from K14Cre;TRF2f/f;Terc-/- tumors, these cells were tumorigenic and formed poorly differentiated SCC (Figure 7D). Itgav+ cells were identified in transplanted tumors by immunofluorescence microscopy (Figure 7E) and comprised 3.2% of cells in these cancers as shown by flow cytometry (Figure 7F). Serial transplantation of Itgav+ tumor cells failed to generate CD34+ or Lgr6+ cancer cells as shown by flow cytometry (Figure 7G, 7H). These results indicated that K14Cre;TRF2f/f;Terc-/- cancers contained a novel Itgav+ self-replicating tumor initiating cell population with restricted differentiation capacity.

**Figure 8:**
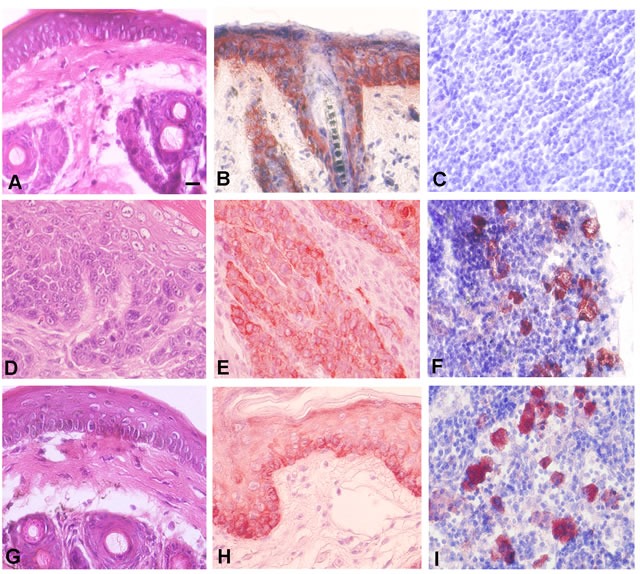
Metastatic cancer cells migrate to regional lymph nodes prior to detection of primary SCC **A.** Epidermis from control mouse as shown by H&E staining. **B.** Expression of the stratified squamous epithelial marker keratin 5 in epidermis from control mouse is shown by immunohistochemistry. **C.** Serial sectioned lymph nodes from control mouse exhibits no evidence of keratin 5 expressing cells. **D.** Epidermis from mouse treated for 16 weeks with DMBA exhibits incipient invasive SCC as shown by H&E staining. **E.** SCC cells from mouse epidermis treated for 16 weeks with DMBA express keratin 5. **F.** Serial sectioned lymph node from mouse epidermis treated for 16 weeks with DMBA exhibits keratin 5 expressing metastatic cells. **G.** Mouse epidermis treated with DMBA for 14 weeks exhibits no evidence of invasive tumor. **H.** Mouse epidermis treated with DMBA for 14 weeks expresses keratin 5. **I.** Serial sectioned lymph node from mouse epidermis treated for 14 weeks with DMBA exhibits keratin 5 expressing metastatic cells. Representative photomicrographs are shown. Scale bar = 10 μm.

In order to understand the relationship between EMT in primary SCC and terminal differentiation of metastatic cells, we hypothesized that metastatic cells may migrate from the primary tumor early in carcinogenesis. To address this possibility, we performed tumor induction time course experiments using keratin 5 expression as a marker of stratified squamous epithelial cells. Control epidermis exhibited keratin 5 expression as predicted (Figure 8A, 8B). Serial sectioning revealed no keratin 5+ cells in regional lymph nodes in control treated mice (Figure 8C). Mouse epidermis treated with DMBA for 16 weeks developed incipient SCC as shown in Figure 8D. These SCCs also expressed keratin 5 protein as shown in Figure 8E. Even at the incipient tumor stage, we detected keratin 5 positive cells in regional lymph nodes of all mice (Figure 8F). We also treated mouse epidermis with DMBA for 14 weeks at which time there was no gross or histopathologic evidence of SCC (Figure 8G). Epidermis treated with DMBA for 14 weeks expressed keratin 5 protein as expected (Figure 8H). Surprisingly we detected keratin 5+ cells in regional lymph nodes from epidermis treated with DMBA for 14 weeks (Figure 8I). These results indicate that metastatic cells can evolve early during carcinogenesis, without histopathologic evidence of primary SCC, and therefore avoid additional genetic damage due to carcinogen exposure in the primary tumor microenvironment which may lead to EMT.

## DISCUSSION

An important finding of our study is the evolution of metastatic clones early in carcinogenesis. We detected metastatic cells in regional lymph nodes prior to histopathologic evidence of primary tumor, suggesting that some stratified epithelial cells transform early during carcinogenesis and are primed for metastasis. Our results indicate that telomere DNA damage signaling does not halt migration of metastatic cells, but induces their terminal differentiation in the lymph node microenvironment. The absence of EMT phenotype in metastatic lymph nodes suggests that EMT may be a late event in tumorigenesis. While not ruled out, it seems less likely that metastatic cells undergo EMT, migrate to the lymph node, reverse the EMT phenotype (mesenchymal-epithelial transition), and then terminally differentiate. Our results contrast with a prevailing model of metastasis in which clones develop slowly within a primary tumor [39]. Our study may help to explain cancer recurrence in the absence of clinical metastasis on initial presentation, and occult primary cancers which present with metastatic lesions but undetectable primary tumors [40–42]. Clinical case reports indicate that approximately 5% of metastatic cancer presents without a detectable primary tumor, and histopathologically confirmed primary tumors have been reported to develop after diagnosis of metastasis [43, 44]. These studies have proposed tumor regression or metastatic dormancy to explain failure to detect primary tumor when metastatic cells are present. Given that tumorigenesis can be closely monitored in the experimental setting, we propose that cancer stem cells may migrate from the primary site prior to producing a clinically detectable tumor. Further studies are needed to characterize the genetics and biology of these metastasis initiating cancer stem cells.

One of the most dramatic findings of our study was the high degree of genomic instability in TRF2/Terc double null SCC. Our model showed severe telomere shortening and aneuploidy which correlated with EMT and expansion of a novel cancer stem cell population. It may be interesting in future studies to determine the extent to which telomere independent effects regulate this phenotype using the Tert null mutant mouse. A previous study showed that TRF2 overexpression in epidermis (K5-TRF2 mice) resulted in short telomeres in the presence of telomerase activity due to recruitment of XPF nuclease activity leading to premature aging and increased cancer [45]. Loss of telomerase activity in K5-TRF2 mice accelerated epithelial carcinogenesis by increased DNA damage and chromosomal instability [46]. Epidermal skin cell dysfunction in these mice was rescued by p53 deletion but skin carcinogenesis was accelerated due to diminished p21 induction [47]. Telomere shortening also has been associated with chromosomal instability in esophageal cancer [48]. Telomere shortening and fusions contribute to disease progression in chronic lymphocytic leukemia with evidence of large scale genomic rearrangements in telomeric regions [49]. Telomere fusions were detected in early breast cancers but not in normal tissue [50]. These studies illustrate the importance of telomere DNA damage response in cancer progression.

Our previously published study demonstrated that loss of telomerase activity inhibited metastasis in a mouse model of SCC [38] but failed to induce terminal differentiation. Despite inhibition, this model developed progressive metastatic lesions indicating that loss of telomerase activity was not sufficient to completely block metastasis. Our present study demonstrated that telomerase deficiency in the context of TRF2 mediated telomere DNA damage response induced terminal differentiation of metastatic SCC. We demonstrated significantly elevated telomere DNA damage signaling and cellular apoptosis in mouse epidermis prior to DMBA treatment, suggesting that the magnitude of the DNA damage response is critical to the terminally differentiated metastatic phenotype. These results may explain lack of clinical response in solid tumor trials of the telomerase inhibitor imetelstat.

Our data demonstrated that CD34+ and Lgr6+ cancer stem cells activate the ALT pathway in the absence of TRF2 expression and telomerase activity. Our previously published study demonstrated that basal cells of epidermis express low levels of ALT activity [51]. These results indicate telomere DNA damage response can activate the ALT pathway in cancer stem cells, providing additional mechanisms of telomere maintenance and cellular survival. A previous study demonstrated long telomeres in ultraviolet radiation induced XPC/Terc deficient epidermal tumors which were attributed to ALT activation [52]. In our present study, DMBA-induced CD34+ and Lgr6+ cancer stem cells were characterized by extensive telomere shortening despite ALT activation. These results suggest that telomere length variation observed in some sarcoma cells is not specific to the ALT pathway, but may result from telomere DNA damage response or carcinogen exposure.

Our results demonstrated that loss of TRF2 expression and telomerase activity resulted in severe depletion of known CD34+ and Lgr6+ epidermal stem cell populations. This depletion was even greater in primary SCC from these double null mutant mice, which exhibited both molecular and cellular characteristics of EMT. Despite their significant depletion, CD34+ and Lgr6+ cancer stem cells were tumorigenic following transplantation. These results contrasted with cancer stem cell expansion observed in other EMT models [33, 52], and our previously published results which did not detect EMT in TRF2 null or Terc null SCC [37, 38]. We characterized a new cancer stem cell population in TRF2/Terc double null SCC characterized by increased integrin α_v_ expression. These cells were tumorigenic on serial transplantation and recapitulated the histopathologic phenotype of poorly differentiated SCC. These integrin α_v_ expressing cells represented a distinct population from other cancer stem cell populations, including significantly reduced CD34 and Lgr6 expression and failure to give rise to these known cancer stem cell populations. Additional cancer stem cell populations have been characterized in human epidermal SCC, including keratin 19, aldehyde dehydrogenase 1, Oct4, and Bmi1 [54, 55]. Lineage analysis and comprehensive gene expression studies will be required to determine the identities of and characterize the relationships between these putative cancer stem cell populations.

Conditional TRF2/Terc double null mutant mice exhibit distinct bending of the tail which is reminiscent of the *crinkled* mouse phenotype [56]. Stem cells of the hair follicle bulge were required for optimal lateral expansion of epidermis during growth of the tail suggesting that this population may inhibit epidermal proliferation resulting tail crinkling. Given that the keratin 14 promoter targets the follicular stem cell populations, telomere DNA damage response and resultant apoptosis in these cells may inhibit epidermal proliferation during rapid growth of the tail in neonatal development.

Our results demonstrate that telomere DNA damage signaling regulates cancer stem cell evolution and metastasis. The results of our study indicate the need for increased scrutiny of cancer diagnostic workups in order to detect micrometastatic disease and improve clinical outcomes for patients.

## MATERIALS AND METHODS

### Mouse breeding and procedures

Mouse strains and experimental procedures were approved by the institutional animal care committee. The Tg(KRT14-Cre)1Amc/J, B6;129P2-*Terf2*^tm1Tdl^/J, and B6.Cg-*Terc*^Tm1Rdp^/J mutant mouse strains were purchased from The Jackson Laboratory (Bar Harbor, ME). Mice were backcrossed to create K14Cre;TRF2f/f;Terc-/- offspring (*n* = 20 for each group). K14Cre;TRF2+/+;Terc+/+ mice of the same background were used as the control group. Single null mutant K14Cre;TRF2f/f;Terc+/+ and K14Cre;TRF2+/+;Terc-/- mice also were analyzed. Mice received 25 μg dimethylbenzanthracene in ethanol applied topically to the epidermis twice weekly [37–38]. Control mice received ethanol vehicle only. The latency, number, and volume of tumors were recorded for each animal. Twenty-two and 24 tumors were analyzed in control and experimental groups respectively. Average tumor volumes were 247 and 235 mm^3^ in control and experimental groups which were not statistically different. Skin and tumors from 9 month old mice were fixed in 4% buffered formaldehyde, flash frozen and stored at -80^0^ C, or trypsin dissociated and cryopreserved in liquid nitrogen. Trypsin dissociated cancer cells also were grown in monolayer culture in Dulbecco's modified Eagle medium, 10% fetal bovine serum, and 40 μg/ml gentamicin at 37^0^ C in a humidified atmosphere of 5% CO_2_.

### qRT-PCR

RNA was extracted from mouse skin and reverse transcribed according to manufacturer's instructions (Invitrogen, Carlsbad, CA, USA). cDNA was amplified using mTRF2 primers 5′- ACTAGCTTACGGAGTCTGC -3′ and 5′-AAGGGGGAGTTTCAGGAGAG -3′. β-actin was amplified using primers 5′-AAAAGCCACCCCCACTCCTAAG-3′ and 5′- TCAAGTCAGTGTACAGGCCAGC-3′ at 94°C for 25 seconds, 55°C for 1 minute, and 72°C for 1 minute. Quantitative PCR was performed using the StepOnePlus system (ThermoFisher Scientific, Waltham, MA, USA).

### Telomeric repeat amplification protocol

The TRAP assay was performed as described previously [38].

### Western blot

Protein was extracted from mouse epidermis and tumors in 1x Laemmli buffer. 75 μg total cellular protein was separated by SDS-PAGE. Proteins were electroblotted to PVDF membranes. Blots were incubated with antibodies to TRF2, phospho-ATM, total ATM, Chk2, p53, or β-actin for 16 hours at 4°C. After washing, blots were incubated for 30 minutes at room temperature with anti-IgG secondary antibody conjugated to horseradish peroxidase. Bands were visualized by the enhanced chemiluminescence method, normalized to β-actin expression, and quantitated by densitometry.

### Telomere length analysis of stem cells

Extreme stem cell depletion in null mutant mice resulted in insufficient genomic DNA for telomere length analysis by Southern blot. We used a quantitative PCR method to measure average telomere length ratios of sorted CD34+ and Lgr6+ stem cells from mouse skin and tumors as described previously [57].

### Fluorescence *in situ* hybridization, immunofluorescence, and immunohistochemistry

Fixed mouse skin and tumor tissue was dehydrated in ethanol, cleared in xylene, and embedded in paraffin. Sections were deparaffinized and stained with hematoxylin and eosin. For telomeric fluorescence *in situ* hybridization, deparaffinized tissue sections or sorted cells were denatured with Cy3 labeled telomeric peptide nucleic acid probe (TTAGGG)_3_ in 70% formamide at 80°C for 10 minutes, followed by overnight incubation at room temperature. After washing, sections and cells were blocked with 10% normal serum and incubated with anti-53BP1 antibody overnight at room temperature. After washing, sections were incubated with anti-IgG secondary antibody conjugated to AlexaFluor 488. After washing, colocalized DNA damage and telomere signals were visualized by fluorescence microscopy (Zeiss LSM 710 META). In separate experiments, PML protein was localized at telomeres of tumor cells using the same immunofluorescence/FISH protocol. Phospho-ATM, phospho-Chk2, CD34, and Lgr6 proteins were localized in mouse epidermis and tumor tissue using the same immunofluorescence protocol. For immunohistochemical analysis, sections were blocked using 10% normal serum and incubated with anti-PCNA or keratin 14 antibodies overnight at room temperature. After washing, sections were incubated with biotinylated secondary antibody at room temperature for 10 minutes. After additional washing, sections were incubated with streptavidin-conjugated horseradish peroxidase for 10 minutes at room temperature. Antigen-antibody complexes were detected by incubation with peroxide substrate solution containing aminoethylcarbazole chromogen. Data were analyzed by Student's *t*-test.

### Cell death analysis

Epidermis and tumor tissue sections were incubated with terminal deoxynucleotidyl transferase and dUTP-fluorescein for 1 h at 37°C according to manufacturer's recommendations (Roche Applied Sciences, Indianapolis, IN, USA). After washing, apoptotic cells were visualized by fluorescence microscopy. The percent fluorescent cells in 10 random high-power fields was determined by counting. Data were analyzed by t test.

### Fluorescence activated cell sorting

Epidermal keratinocytes and SCCs were dissociated by trypsinization, washed in PBS, and incubated with phycoerythrin conjugated CD34, integrin α_v_, or AlexaFluor 488 conjugated Lgr6 antibodies. Samples were washed in PBS followed by FACS. Data were analyzed by t test.

### RNA extraction and gene expression profiling

RNA extraction and gene expression profiling was performed as described previously [38].

### Metaphase chromosomal spreads

Tumor cell cultures were treated with 0.1 μg/ml colcemid for 3 hours at 37°C. Cell pellets were suspended in 60 mM KCl and incubated at room temperature for 30 minutes. After centrifugation, cell pellets were fixed 3 times in 3:1 methanol:acetic acid and spotted onto microscope slides. Cy3 labeled telomeric probe was hybridized to the metaphase spreads overnight at room temperature. After washing, slides were coverslipped with antifade mounting medium containing DAPI and photographed using fluorescence microscopy.

### Chromosome orientation-fluorescence *in situ* hybridization

Tumor cells were incubated for 16 hours with 30 μM 5-bromo-2′-deoxyuridine and 10 μM 5-bromo-2′-deoxycytidine followed by a 90 minute incubation with 0.1 μg/ml colcemid. Harvested cells were incubated in 60 mM KCl at room temperature for 30 minutes, fixed 3 times in 3:1 methanol:acetic acid, and spotted on glass slides. Cells were incubated with 0.5 mg/ml RNase A for 10 minutes at 37°C. Cells were incubated with 0.5 μg/ml Hoechst 33258 for 15 minutes at room temperature. Cells were UV-irradiated for 30 minutes and incubated with 800 U exonuclease III for 10 minutes at room temperature. After washing, slides were dehydrated in ethanol and hybridized with TelG-Cy3 peptide nucleic acid probe in 70% formamide, followed by TelC-FITC probe. Slides were coverslipped using antifade mounting medium containing DAPI.

### Telomeric circular DNA analysis

Genomic DNA extracted from sorted tumor cells was digested with *Hin*fI and *Rsa*I restriction enzymes. Telomeric circular DNA was amplified with φ29 DNA polymerase in the absence of dCTP and blotted to nylon membranes. Blots were hybridized with ^32^P-labeled (TTAGGG)_4_ probe overnight at 50°C. After washing in 4X SCC for 30 minutes at room temperature, blots were exposed to autoradiographic film for 16 hours at −80°C.

### Tumorigenicity experiments

10^2^-10^4^ CD34+, integrin α_v_+, or Lgr6+ sorted cells from primary tumors were injected subcutaneously in Matrigel into 2 month old NU/J mice. Mice were examined weekly for tumor formation for up to 6 months. Five transplanted tumors per group were analyzed; there were no significant differences in tumor volume between each group. Mice underwent complete necropsy; tumors were dissected and portions of each were fixed in 4% buffered formaldehyde for microscopy or dissociated for flow cytometry.

### Antibodies

TRF2 Santa Cruz Biotechnology SC-9143, α-actin Novus NB600-303, 53BP1 Novus NB100-304, pATM Santa Cruz Biotechnology SC-47739, pChk2 Abcam AB85743, p53 BD Biosciences BD554147, PCNA Santa Cruz Biotechnology SC-7907, CD34 Santa Cruz Biotechnology SC-18917, Lgr6 Santa Cruz Biotechnology SC-48236, K14 Abcam AB7800, p63 Santa Cruz Biotechnology SC8431, vimentin Cell Signaling 5741T, E-cadherin Cell Signaling 3195, Itgav Abcam AB179475, K5 Abcam AB24647

## SUPPLEMENTARY MATERIALS FIGURES


