# Supplementary material for: Telomere DNA damage signaling regulates cancer stem cell evolution, epithelial mesenchymal transition, and metastasis
Source: Oncotarget. 2017 Sep 16;8(46):80139–55. doi: 10.18632/oncotarget.20960 (PMC5655185; doi:10.18632/oncotarget.20960)
Supplement: Supplementary file 1 [file oncotarget-08-80139-s001.pdf]

Telomere DNA damage signaling regulates cancer stem cell evolution, epithelial mesenchymal transition, and metastasis

SUPPLEMENTARY MATERIALS

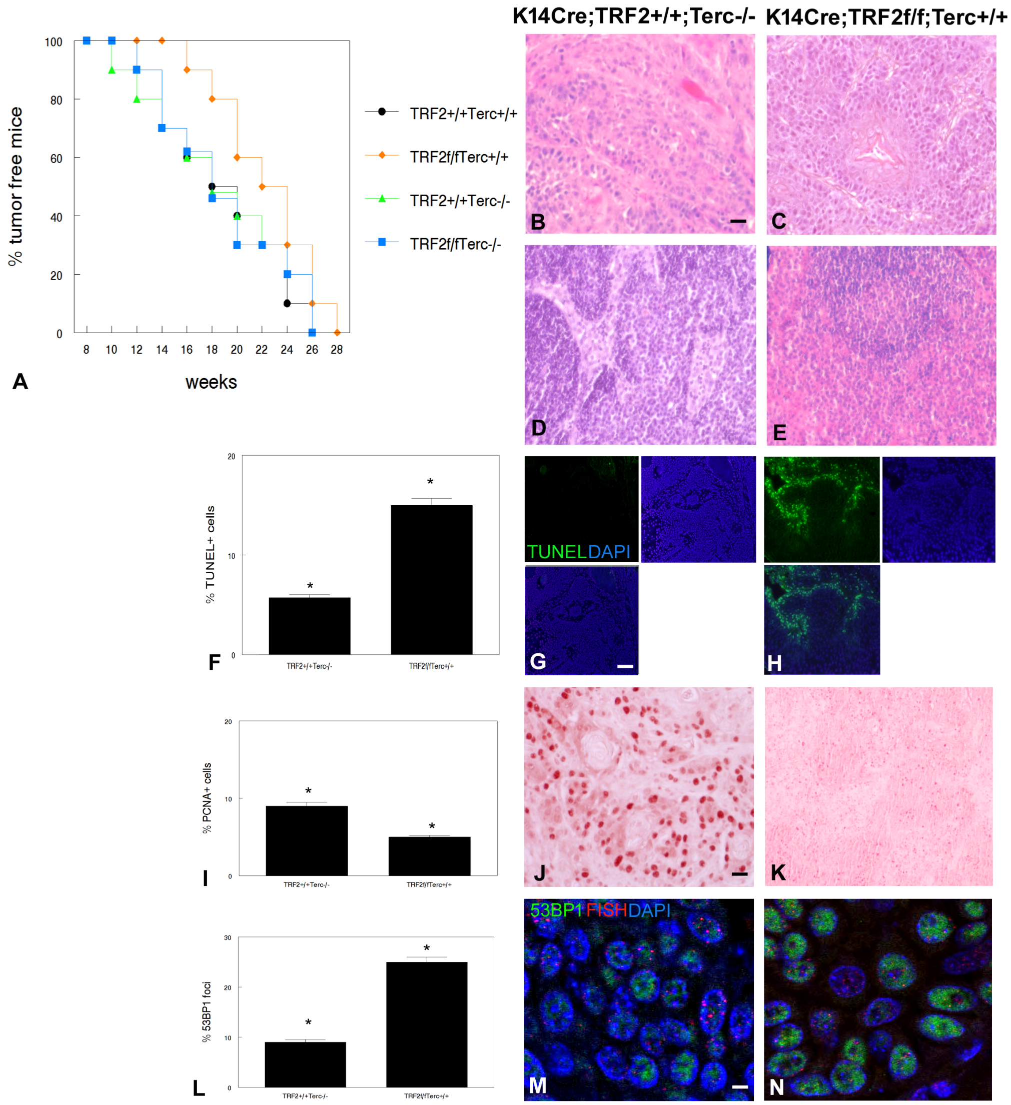

Supplementary Figure 1: Increased latency in K14Cre;TRF2f/f;Terc+/+ SCC. **(A)** Tumor latency of K14Cre;TRF2+/+;Terc+/+, K14Cre;TRF2f/f;Terc+/+; K14Cre;TRF2+/+;Terc-/-, and K14Cre;TRF2f/f;Terc-/- SCC. Percent tumor free mice and weeks tumor latency are shown. Histopathology of primary SCC from K14Cre;TRF2+/+;Terc-/- **(B)** and K14Cre;TRF2f/f;Terc+/+ **(C)** mice is shown by H&E staining. Scale bar = 10 µm. Histopathology of metastatic SCC from K14Cre;TRF2+/+;Terc-/- **(D)** and K14Cre;TRF2f/f;Terc+/+ **(E)** mice is shown by H&E staining. Apoptotic cells in primary SCC from K14Cre;TRF2+/+;Terc-/- **(G)** and K14Cre;TRF2f/f;Terc+/+ **(H)** mice are shown by TUNEL assay and quantitated in **(F)**. Scale bar = 10 µm. Proliferating cells were detected in K14Cre;TRF2+/+;Terc-/- **(J)** and K14Cre;TRF2f/f;Terc+/+ **(K)** primary SCC by PCNA immunohistochemistry and quantitated in **(I)**. Co-localization of 53BP1 (shown by immunofluorescence, AlexaFluor 488) at telomeres (shown by fluorescence in situ hybridization, Cy3) in histopathologic sections from K14Cre;TRF2+/+;Terc-/- **(M)** and K14Cre;TRF2f/f;Terc+/+ **(N)** primary SCC and quantitated in **(L)**. Error bars indicate SEM. Nuclei were counterstained with DAPI. Scale bar = 5 µm.

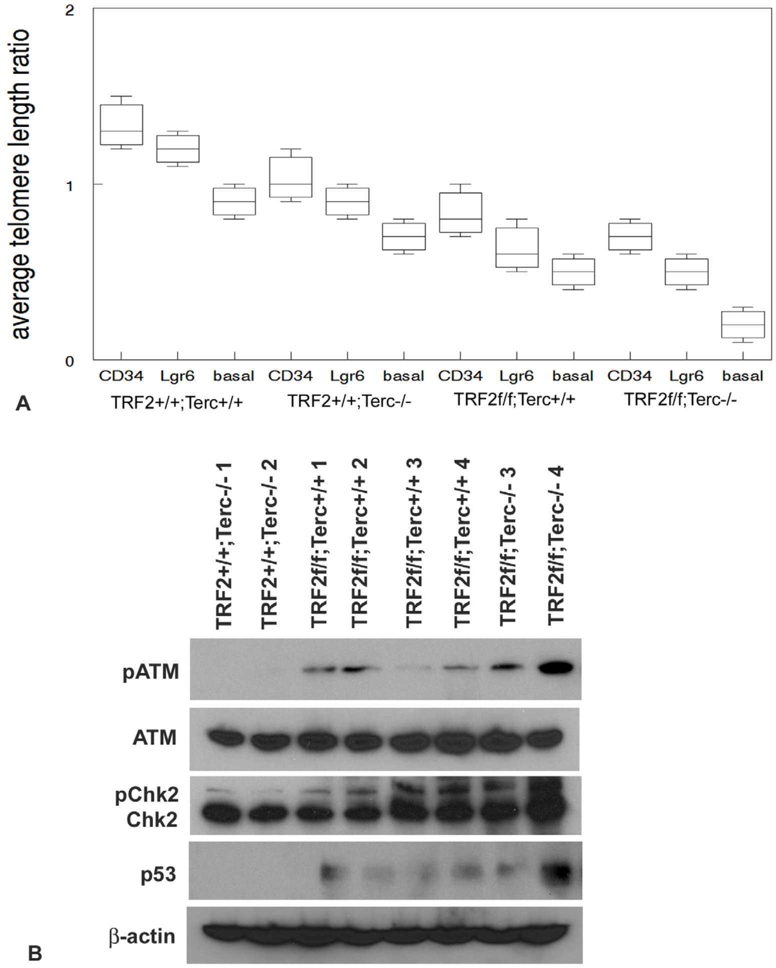

Supplementary Figure 2: (A) Average telomere length ratios in CD34+ stem, Lgr6+ stem, and basal cells from K14Cre;TRF2+/+;Terc+/+, K14Cre;TRF2+/+;Terc-/-, K14Cre;TRF2f/f;Terc+/+, and K14Cre;TRF2f/f;Terc-/- primary SCC were determined by qPCR. Error bars represent SEM. **(B)** Phospho-ATM, phospho-Chk2, and p53 expression in K14Cre;TRF2+/+;Terc-/-, K14Cre;TRF2f/f;Terc+/+, and K14Cre;TRF2f/f;Terc-/- primary SCC is shown by western blot. β-actin expression was used to control for equal loading of each lane. Representative blots are shown.

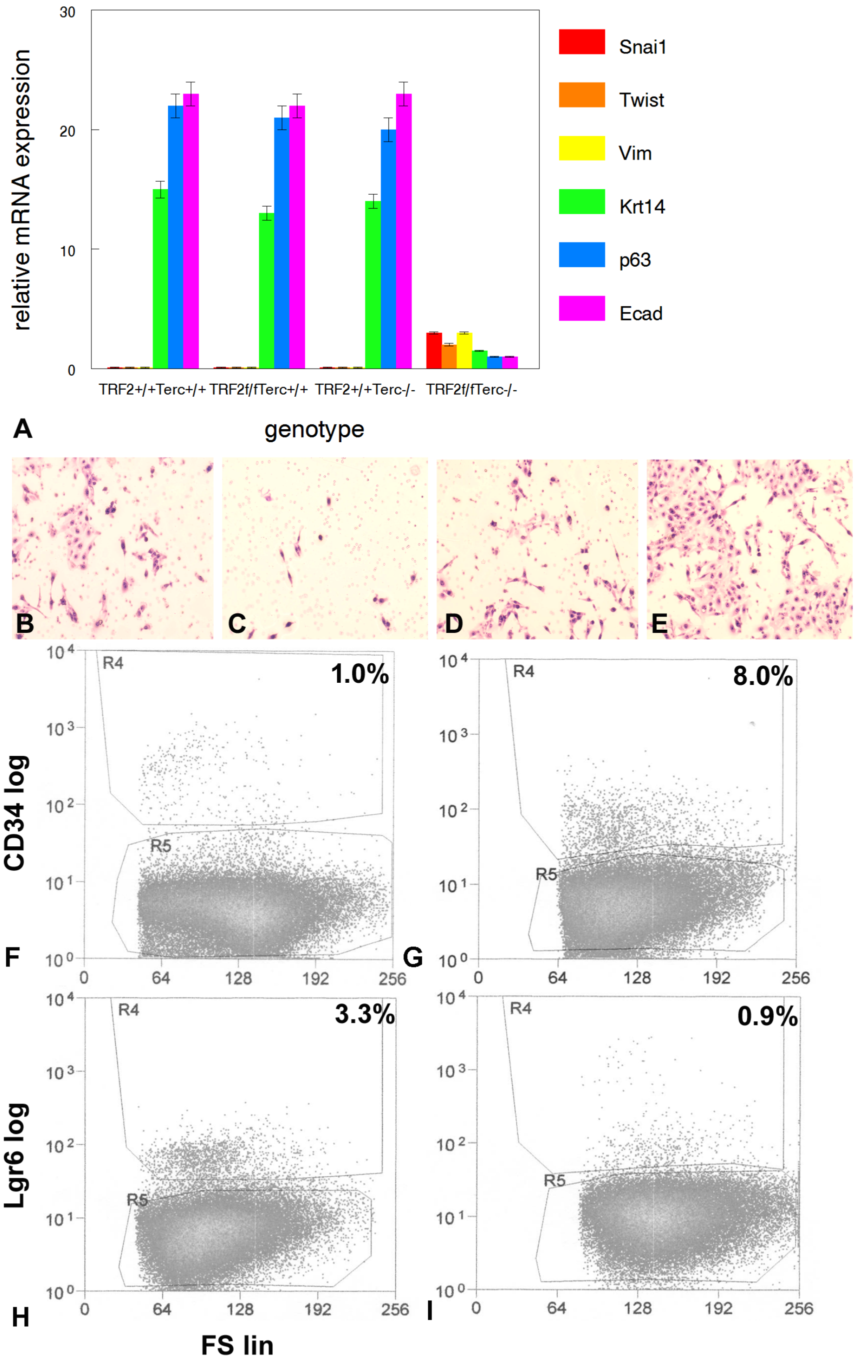

Supplementary Figure 3: (A) Expression of EMT (Snai1, Twist, Vim) and epithelial differentiation (Krt14, p63, Ecad) mRNAs in K14Cre;TRF2+/+;Terc+/+, K14Cre;TRF2f/f;Terc+/+, K14Cre;TRF2+/+;Terc-/-, and K14Cre;TRF2f/f;Terc-/- SCC**.** In vitro invasion analysis using cancer cells from K14Cre;TRF2+/+;Terc+/+ **(B)**, K14Cre;TRF2+/+;Terc-/- **(C)**, K14Cre;TRF2f/f;Terc+/+ **(D)**, and K14Cre;TRF2f/f;Terc-/-**(E)** genotypes. FACS analysis of CD34+ cancer stem cells from K14Cre;TRF2+/+;Terc-/- **(F)** and K14Cre;TRF2f/f;Terc+/+ **(G)** SCC. FACS analysis of Lgr6+ cancer stem cells from K14Cre;TRF2+/+;Terc-/- **(H)** and K14Cre;TRF2f/f;Terc+/+ **(I)** SCC. Fluorescence is shown by log scale (y axis) and forward scatter is shown by linear scale (x axis). The percent positive cells in each group are shown.

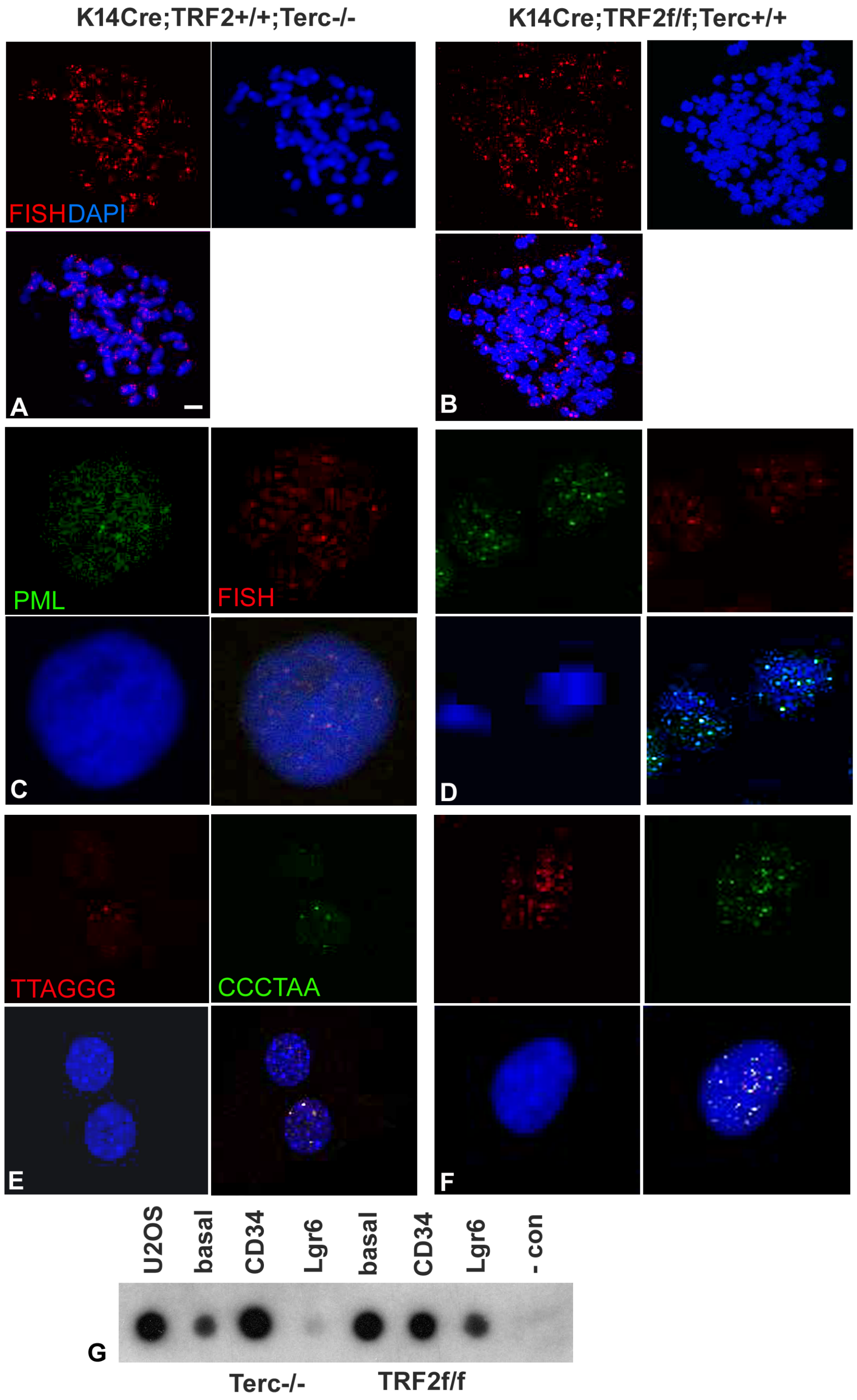

Supplementary Figure 4: TRF2 and Terc null mutations induce chromosomal instability and ALT in primary SCC. (A) Telomeres are shown by FISH (Cy3) on metaphase chromosomal spreads of SCC cells from K14Cre;TRF2+/+;Terc-/- (A) and K14Cre;TRF2f/f;Terc+/+ **(B)** mice. Chromosomes were counterstained with DAPI. Scale bar = 2 µm. APBs (PML protein shown by immunofluorescence, AlexaFluor 488) at telomeres (shown by fluorescence in situ hybridization, Cy3) in K14Cre;TRF2+/+;Terc-/- **(C)** and K14Cre;TRF2f/f;Terc+/+ **(D)** primary tumors are shown. Nuclei were counterstained with DAPI. ALT-associated sister chromatid exchange in K14Cre;TRF2+/+;Terc-/- **(E)** and K14Cre;TRF2f/f;Terc+/+ **(F)** SCC cells is shown by CO-FISH. Co-localization of DNA strand specific signals (Cy3, FITC) indicate sister chromatid exchange. Representative photomicrographs are shown. **(G)** Telomeric circular DNA in sorted CD34+, Lgr6+, and CD34-Lgr6- basal cells from K14Cre;TRF2+/+;Terc-/- and K14Cre;TRF2f/f;Terc+/+ SCC. U2OS cells were used as the positive control, and reactions without genomic DNA or polymerase were used as the negative control.
